# Kinetic Modeling and Graphical Analysis of 18F-Fluoromethylcholine (FCho), 18F-Fluoroethyltyrosine (FET) and 18F-Fluorodeoxyglucose (FDG) PET for the Fiscrimination between High-Grade Glioma and Radiation Necrosis in Rats

**DOI:** 10.1371/journal.pone.0161845

**Published:** 2016-08-25

**Authors:** Julie Bolcaen, Kelly Lybaert, Lieselotte Moerman, Benedicte Descamps, Karel Deblaere, Tom Boterberg, Jean-Pierre Kalala, Caroline Van den Broecke, Filip De Vos, Christian Vanhove, Ingeborg Goethals

**Affiliations:** 1 Department of Nuclear Medicine, Ghent University Hospital, Ghent, Belgium; 2 iMinds-IBiTech-MEDISIP, Department of Electronics and Information Systems, Ghent University, Ghent, Belgium; 3 Department of Radiology, Ghent University Hospital, Ghent, Belgium; 4 Department of Radiation Oncology, Ghent University Hospital, Ghent, Belgium; 5 Department of Neurosurgery, Ghent University Hospital, Ghent, Belgium; 6 Department of Pathology, Ghent University Hospital, Ghent, Belgium; 7 Department of Radiopharmacy, Ghent University, Ghent, Belgium; Wayne State University, UNITED STATES

## Abstract

**Background:**

Discrimination between glioblastoma (GB) and radiation necrosis (RN) post-irradiation remains challenging but has a large impact on further treatment and prognosis. In this study, the uptake mechanisms of 18F-fluorodeoxyglucose (18F-FDG), 18F-fluoroethyltyrosine (18F-FET) and 18F-fluoromethylcholine (18F-FCho) positron emission tomography (PET) tracers were investigated in a F98 GB and RN rat model applying kinetic modeling (KM) and graphical analysis (GA) to clarify our previous results.

**Methods:**

Dynamic 18F-FDG (GB n = 6 and RN n = 5), 18F-FET (GB n = 5 and RN n = 5) and 18F-FCho PET (GB n = 5 and RN n = 5) were acquired with continuous arterial blood sampling. Arterial input function (AIF) corrections, KM and GA were performed.

**Results:**

The influx rate (K_i_) of 18F-FDG uptake described by a 2-compartmental model (CM) or using Patlak GA, showed more trapping (k_3_) in GB (0.07 min^-1^) compared to RN (0.04 min^-1^) (p = 0.017). K_1_ of 18F-FET was significantly higher in GB (0.06 ml/ccm/min) compared to RN (0.02 ml/ccm/min), quantified using a 1-CM and Logan GA (p = 0.036). 18F-FCho was rapidly oxidized complicating data interpretation. Using a 1-CM and Logan GA no clear differences were found to discriminate GB from RN.

**Conclusions:**

Based on our results we concluded that using KM and GA both 18F-FDG and 18F-FET were able to discriminate GB from RN. Using a 2-CM model more trapping of 18F-FDG was found in GB compared to RN. Secondly, the influx of 18F-FET was higher in GB compared to RN using a 1-CM model. Important correlations were found between SUV and kinetic or graphical measures for 18F-FDG and 18F-FET. 18F-FCho PET did not allow discrimination between GB and RN.

## Introduction

Differentiating tumor recurrence form radiation necrosis (RN) during follow-up of glioblastoma (GB) patients post-treatment remains challenging. The incidence of RN in GB patients was reported to be 30% and correct diagnosis has a large impact on further treatment and prognosis [1]. The primary mechanisms of RN are vascular endothelial injury or damage to oligodendroglia [2]. Because of the disruption of the blood brain barrier (BBB) in both GB and RN, contrast enhancement is usually present on both MRI and CT. Therefore these entities cannot be distinguished based on conventional CT or MRI only. In GB, increased membrane turnover, vascularity and cellularity found by MR spectroscopy (MRS), perfusion, and diffusion should lead to suspicion of a tumor [1]. However, in most institutions these techniques are not used routinely. PET is also promising, possibly able to visualize differences in metabolic activity between GB and RN. Multiple studies investigated the potential of 18F-fluorodeoxyglucose (18F-FDG) in discriminating RN from tumor, however, equivocal results have been published [3–6]. Pöpperl et al. confirmed that recurrence had focal 18F-fluoroethyltyrosine (18F-FET) uptake with significantly higher uptake compared to non-recurrence [7]. A major advantage of 18F-FET is the lack of uptake in inflammatory tissue [8]. Furthermore, 18F-fluoromethylcholine (18F-FCho) was suggested to be promising in separating RN form tumor recurrence [9,10,11]. At present, 18F-FCho is mainly used for restaging of prostate cancer in case of biochemical relapse [12]. Degrado et al. introduced 18F-FCho for brain tumor imaging [13]. The potential of 18F-FDG and 18F-FET using standard uptake values (SUV) was confirmed in our previous *in vivo* study. Using 18F-FCho PET, uptake in GB was not significantly different from RN [9]. SUV is the most widely used parameter for PET analysis but do not take into account possible influences on quantification by blood volume and metabolite formation [12,14]. Therefore, our goal was to characterize and further clarify the mechanism of uptake of 18F-FDG, 18F-FET and 18F-FCho in GB and RN quantitatively using kinetic modeling (KM), as already described in [9]. For 18F-FDG, a two tissue compartmental model with one input function (2C1i) was proposed [15,16] and suggested to be useful for differentiating high-grade glioma from brain lymphoma [17]. Our hypothesis suggests a higher k3 in GB compared to RN. Also Patlak graphical analysis (GA) has been proposed for analyzing 18F-FDG PET [14,18,19]. Quantification of 18F-FET uptake, mediated by large amino acid transporters (LAT), B^0+^ and B^0^ transport mechanisms [8], has been shown to be feasible using Logan GA and one tissue compartment model analysis (1C1i) [20]. The only assumptions in applying Logan analysis were reversible binding of the tracer and linearity of the given expressions, which are met for FET [14,18,20]. Assuming a higher amount of amino acid transport mechanisms in tumor, K_1_ is hypothesized to be higher in GB than in RN. The kinetic model for 18F-FCho is not clear because of its oxidative metabolism to 18F-Fluorobetaine (18F-FBet) [12,20,21,22]. Therefore in this study, next to GA, three compartmental models were evaluated, including 1C1i, 2C1i and a model using three compartments containing two input functions (18F-FCho and 18F-FBet) (3C2i).

## Materials and Methods

### F98 GB model

**Fig 1:**
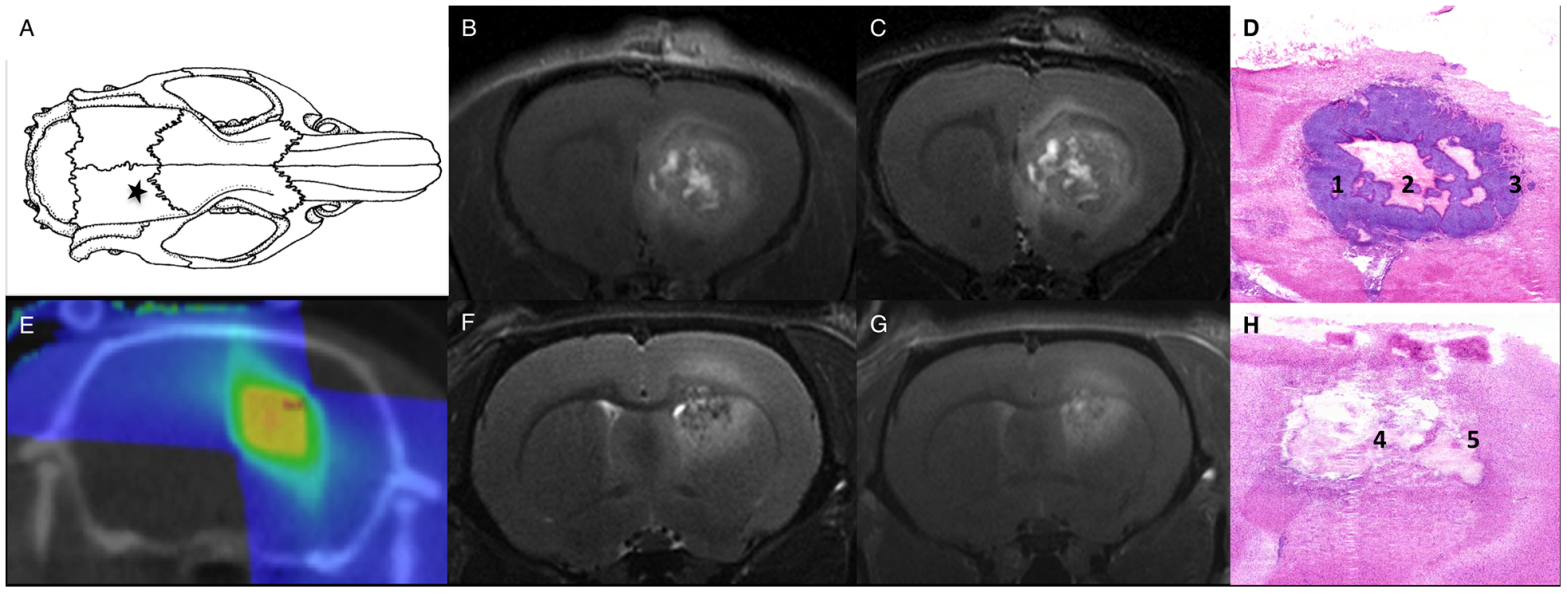
GB and RN model. Location for inoculation of F98 glioblastoma cells (★A), glioblastoma T2-weighted (B) and T1-weighted contrast-enhanced (C) MRI, hematoxylin and eosin staining (D) confirms the presence of glioblastoma tumor cells (1), central tumor necrosis (2) and abundant blood vessels in the perinecrotic tumor rim (3), dose plan for radiation therapy inducing RN (E), radiation necrosis T2-weighted (F) and T1-weighted contrast-enhanced (G) MRI and hematoxylin and eosin staining (H) confirming a large necrotic area with profound vascular changes (4), surrounded by a rim of macrophages (5).

Our GB rat model was optimized previously (Fig 1A–1D) [9]. F98 GB cells, obtained from ATCC^®^ (CRL-2397), were inoculated in the right frontal hemisphere (Fig 1A) of 39 female Fischer F344 rats (Charles River^®^) (body weight 177±8 g). The rats were anesthetized with ketamine/xylazine (4/3; 0.13 ml/100 g). Post-surgery, a close follow-up of the animals was performed (body temperature, wound healing and behavior). Animals were kept separately post-inoculation.

### RN model

Induction of RN in normal brain tissue in a second group of 34 female Fischer F344 rats (Charles River^®^) (209±39 g) was achieved using the small animal radiation research platform (SARRP, Xstrahl^®^, Surrey, UK). Conformal arc micro-irradiation was optimized previously [23]. 3 arcs and a 3x3 mm collimator were used for the delivery of 60 Gy in a single dose (Fig 1E). RN was confirmed when hyperintense signal on T2-weighted MRI and contrast-enhancement on T1-weighted MRI was visible (Fig 1F and 1G).

All animals were kept under environmentally controlled conditions (12-h normal light/dark cycles, 20°C–24°C, and 40–70% relative humidity) with food and water ad libitum. Follow-up of all animals was done by monitoring their body weight, food, water intake and their activity and normal behavior. The method of euthanasia was a lethal dose of pentobarbital sodium (160 mg/kg). Euthanasia was performed prior to the experimental endpoint if a decline of 20% body weight was observed or when the normal behavior severely deteriorated (e.g. lack of grooming). The study was approved by the Ghent University Ethical Committee on animal experiments (ECD12/28-A2).

### Multimodality imaging to localize GB and RN

#### MRI

Eight days post-inoculation (p.i.) MRI was performed using a 7 Tesla micro-MR system (PharmaScan 70/16, Bruker BioSpin, Ettlingen, Germany) to confirm tumor growth in the GB group. Follow-up of tumor growth was done twice a week p.i. using MRI. Previous optimization of the RN model showed no lesion earlier than 5 months post-irradiation. Therefore, in the RN group, longitudinal MRI was performed weekly to detect RN, starting 5 months post-irradiation. For MRI protocol details, see [9]. Fig 1 shows T2- and contrast enhanced T1-weighted MRI of GB and RN. In the GB group, in case of confirmed tumor growth on day eight p.i., PET with arterial blood sampling (ABS) and MRI for tumor delineation were acquired on day 14–16 p.i.. In the RN group, in case of a confirmed contrast-enhancing RN lesion on MRI, PET was acquired within the week.

#### Dynamic PET with ABS

**Fig 2:**
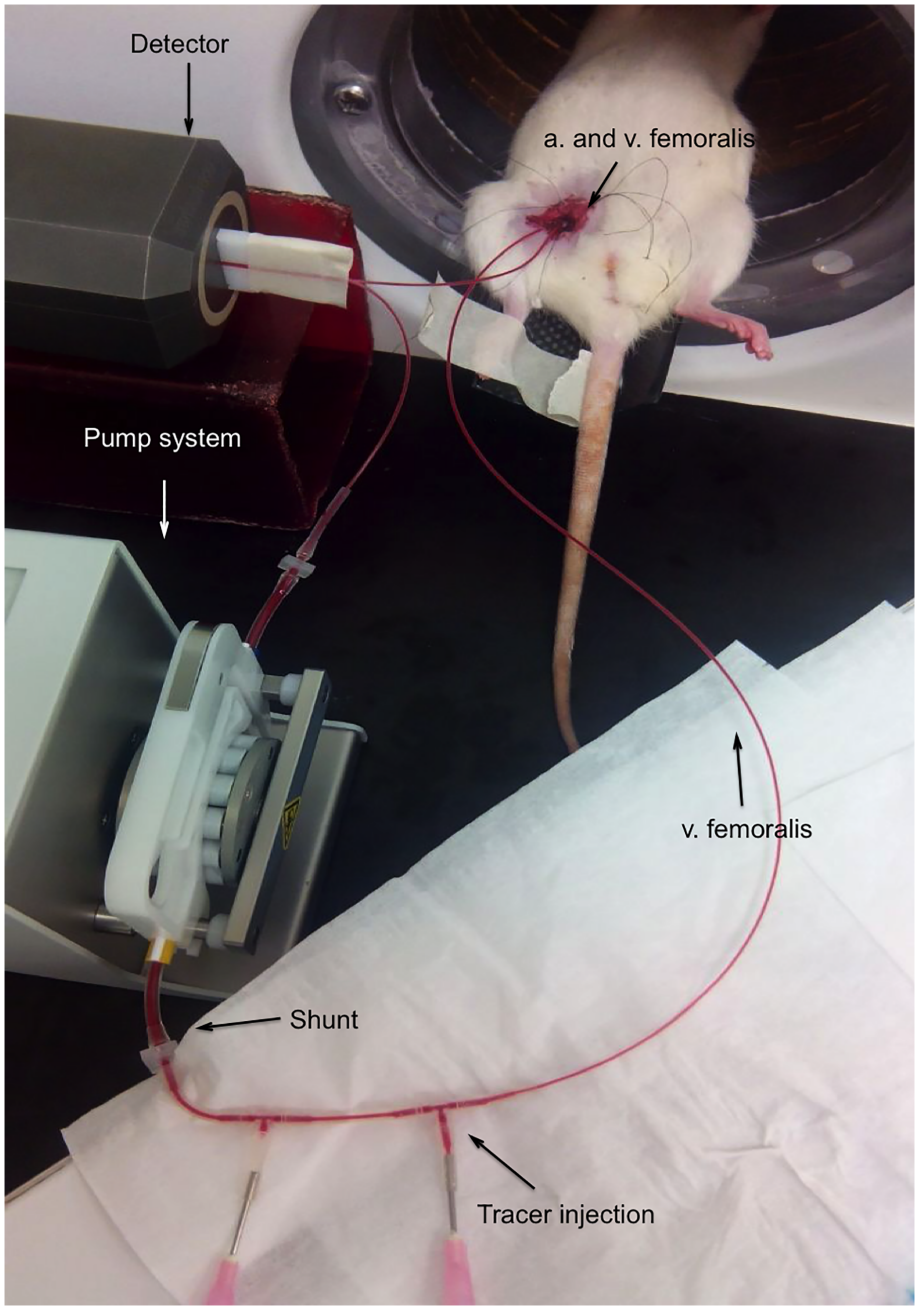
Arterial blood sampling set-up. The micro-PET system (FLEX Triumph II, TriFoil Imaging^®^, Northridge CA) was used. At a distance of 10 cm the arterial catheter was fixed and inserted in the detector of the Twilite system (Swisstrace GmbH, Switserland). Using a shunt and a pump system a continuous blood flow was enabled (200 μl/min). Data acquisition was performed with PMOD (3.405, PMOD technologies^®^, Zürich, Switserland). The system was calibrated weekly.

Catheterization. A catheter was placed in the arteria (a.) femoralis and vena (v.) femoralis measuring the whole blood AIF without blood loss (Fig 2). Rats were anesthetized with 2% isoflurane mixed with oxygen (0.3 L/min). Femoral vein, artery and nerve were separated (microscope, Optika^®^). The distal end of the vena/arteria was bound and blood flow was blocked. Using a 25G (BD^®^) needle an orifice was made to insert a 40 cm PE50 tubing filled with heparine solution (50 U/ml).

PET. Dynamic list mode acquisitions of 20 min for 18F-FCho (GB n = 5 and RN = 5), 55 min for 18F-FET (GB n = 5 and RN = 5) and 60 min for 18F-FDG PET (GB n = 6 and RN = 5) were acquired (37 MBq). In addition, a 30-min late 18F-FDG PET static scan was acquired 240 min p.i.. All scans were reconstructed into a 200x200x64 matrix by a 2D Maximum Likelihood Expectation Maximization algorithm (LabPET 1.12.1, TriFoil Imaging^®^, Northridge CA) using 60 iterations and a voxel size of 0.5x0.5x1.157 mm. No partial volume corrections were performed. Time frames for 18F-FDG scans were 12x10s/6x20s/6x1min/10x2min/6x5min, for 18F-FCho 12x10s/6x20s/6x1min/2x5min and for 18F-FET 12x10s/6x20s/6x1min/10x2min/5x5min.

### PET quantification

#### Semi-quantitative analyses

Details on SUV calculation can be found in our previous publication [9]. The uptake in the last 20 min (18F-FDG and 18F-FET) or the last 10 min (18F-FCho) was selected to calculate lesion-to-normal tissue ratios (LNRs), analogous to [9]. LNR_mean_ is defined as the ratio of SUV_mean_ in the tumor or RN VOI to SUV_mean_ in the reference VOI. LNR_max_ is defined as the ratio of SUV_max_ in the tumor or RN VOI to SUV_mean_ in the reference VOI. Cubic VOIs of 3 × 3 × 3 mm located in the contralateral occipital region were used as a reference.

#### Quantitative analyses

Calibration factor, dispersion, decay, and delay AIF correction. The calibration factor was calculated using the calibration TAC and PET. Decay correction was performed after defining the start time of the acquisition. A delay of 10 s was included. The AIF was corrected for dispersion using the formula of Convert et al. [24,25]:

Ca (t) = g(t) + τdisp x dg/dt

C_a_ (t) was the dispersion corrected blood curve, g(t) was the measured blood time-activity and τ_disp_ was a constant calculated as described by [24].

Plasma-blood ratio. 18F-FDG, plasma-blood ratios were described by Weber et al. [26]. 18F-FET plasma-blood ratios were obtained by collecting blood 1-30-55 min (n = 2) and 5-15-40 min (n = 2) p.i. (37 MBq). 18F-FCho plasma-blood ratios were determined 1-5-15 min p.i. (n = 3).

Metabolite correction. Metabolite correction was required for 18F-FCho due to its fast oxidation in the liver and kidneys (S3 Fig). Metabolites in plasma were determined using the method of Bansal et al. [27]. Aqueous and organic fractions were separated and counted. The aqueous layer (100 μl) was injected to a HPLC column (Alltech^®^, Grace). The HPLC consisted of a Waters 1525 binary pump (Waters, Milford, MA, USA), a Waters Breeze data acquisition, and an Alltima silica NP column (5 μm, 10x250 mm). The eluent was collected in 30-sec fractions and measured using a γ counter (Cobra^®^, Packard Canberra).

#### Kinetic modeling

KM was performed using PMOD selecting 2C1i for 18F-FDG, 2C1i or 1C1i for 18F-FET and 2C1i, 1C1i or 3C2i for 18F-FCho. For FDG a lumped constant (LC) of 0.89 was selected [28] and the plasma glucose level was determined in a rat with 6 h food deprivation. The models were described by micro-parameters: K_1_, k_2_, k_3_ and k_4_ [15]. The influx (K_i_) was calculated as K_1_*k_3_/(k_2_+k_3_) and the phosphorylation rate (K_p_) as k_3_/(k_2_+k_3_). The distribution volume V_d_ (K_1_/k_2_) was quantified when applying 1C1i. Tracer plasma curves were fitted using a three-exponential decay function [22]. Model fitting was optimized by (1) visual inspection, (2) evaluation of standard errors (SE), and (3) goodness-of-fit displayed by the Akaike information criterion (AIC), Schwartz criterion (SC) and model selection criterion (MSC). The AIC criterion estimates an information-theoretic measure, the Kullback—Leibler distance, which quantifies the information lost, if a model is used which only approximates the true model [29]. The Schwartz criterion (SC), also called Bayesian Information Criterion (BIC), attempts to identify a posteriori what the most probable model is for a particular data set. The SC is similar to the AIC but includes an additional penalty for the number of data points and therefore favors simpler models [30]. Another criterion used in the Scientist Software (MicroMath, Saint Louis, Missouri USA) is the Model Selection Criterion (MSC) [31]. The preferred model is the one with the lowest AIC value, the lowest SC value and the highest MSC value. No threshold was applied.

#### Graphical analysis

**Fig 3:**
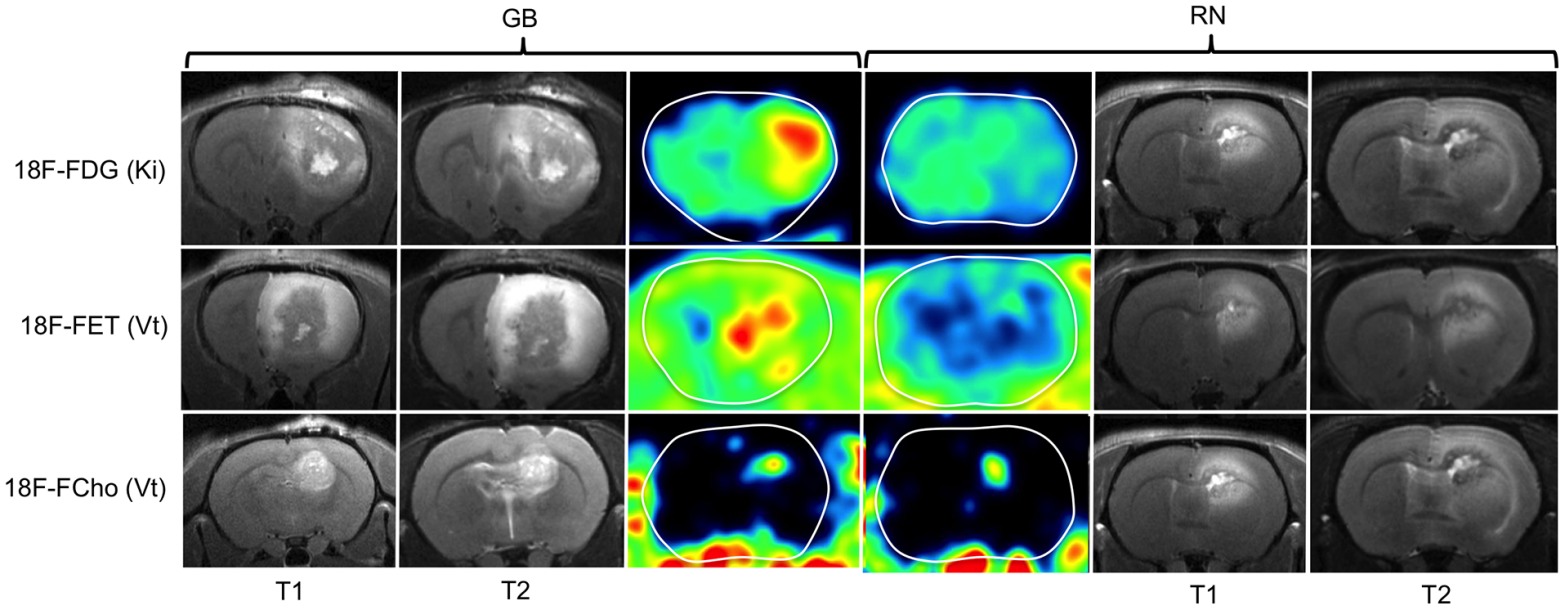
T1- and T2-weighted MRI and parametric maps of the Patlak slope (Ki) and Logan slope (Vt) in GB and RN. Ki of 18F-FDG (row 1) and Vt of 18F-FET (row 2) were higher in GB than in RN. Vt shows heterogeneously increased values both in GB and RN lesions on 18F-FCho PET (row 3).

Patlak regression was applied characterizing 18F-FDG, 18F-FET and 18F-FCho uptake. If the data were consistent with this model, the curve became linear with a slope K_i_ (= K_1_.k_3_/k_2_+k_3_) and an intercept V (distribution volume). Logan GA was applied to the 18F-FET and 18F-FCho data. The slope K represented K_1_/k_2_ for 1C1i and K_1_/(k_2_(1+k_3_/k_4_)) for 2C1i [14,18]. Parametric images of K_i_ and V_t_ were calculated and a Gaussian smoothing filter (FWHM 1*1*1 mm) was applied (Fig 3).

### Statistical analysis

Statistical analysis of the SUV, LNRs, KM and GA derived variables between GB and RN was performed using the Mann-Whitney U non-parametric test. Spearman correlations (R_s_) between SUV variables and kinetic/graphical measures were calculated. A probability value p < 0.05 is considered statistically significant.

## Results

### GB and RN model

GB. Eight days post-inoculation, a contrast-enhancing tumor was visible on MRI in the right frontal region (n = 31). Confirmation MRI was negative in 8 rats. Typical MRI showed hyperintense signal on T2-weighted MRI (Fig 1B) and contrast enhancement on T1-weighted MRI (Fig 1C). On the day of PET imaging, the mean contrast enhancing tumor volume on T1-weighted MRI was 99 ± 73 mm^3^ (n = 16). GB histology was confirmed (Fig 1D) [9,23].

RN. In 15 rats RN was visible on MRI 31±6 weeks post-irradiation and these were included for PET with ABS. If no lesion was visible 34±4 weeks post-RT, they were not included (n = 17). RN was characterized by a heterogeneously hyperintense signal in the center on T2-weighted MRI (Fig 1F), and a heterogeneous hyperintense signal on contrast-enhanced T1-weighted MRI (Fig 1G). On the day of PET imaging, the mean contrast enhancing tumor volume on T1-weighted MRI was 36 ± 20 mm^3^ (n = 15). RN histology was confirmed (Fig 1H) [9].

### PET quantification

#### Semi-quantitative analysis

PET images and SUV analysis are given in S1 Fig.

^18^F-FDG. On the early 18F-FDG scan, mean SUV_max_ (7.68±1.92 for GB and 4.54±1.47 for RN), mean LNR_mean_ (1.5±0.18 for GB and 0.98±0.07 for RN) and mean LNR_max_ (2.62±0.42 for GB and 1.24±0.11 for RN) were significantly lower in RN compared to GB (p = 0.017; p = 0.004 and p = 0.004, respectively). The difference in SUV_max_ (6.59±1.70 for GB and 3.26±0.86 for RN), LNR_mean_ (2.33±0.81 for GB vs. 1.29±0.09 for RN) and LNR_max_ (4.49±0.49 for GB vs. 1.93±0.25 for RN) was higher on the late 18F-FDG PET images than on the PET images reconstructed from the last time frame of the dynamic acquisition (both p = 0.008).

18F-FET. Mean SUV_max_ and SUV_mean_ values are borderline non-significantly different between GB and RN (p = 0.071). The LNR_mean_ (2.15±0.12 for GB and 1.61±0.17 for RN) and LNR_max_ (3.61±0.21 for GB and 2.30±0.34 for RN) were statistically significantly different between GB and RN (p = 0.036).

18F-FCho. Mean SUV_max_ and SUV_mean_ were not significantly different between GB (0.69±0.09 and 0.32±0.08, respectively) and RN (0.87±0.17 and 0.44±0.06 respectively) (p = 0.095). Also the LNR_mean_ and the LNR_max_ were not significantly different between GB and RN (p = 0.095 and p = 0.841, respectively), with slightly lower values for GB (1.49±0.14 and 3.24±0.77, respectively) compared to RN (1.84±0.31 and 3.63±0.76, respectively).

#### Quantitative analysis

Dispersion correction. Based on the catheter internal diameter (span_v_ = 43.5), withdrawal speed (v = 200 μl/min) and distance between animal and detector (d = 10 cm), τ_disp_ was 4.63 s [24]. The original blood curves and their dispersion-corrected blood curves are displayed in S2A Fig.

Plasma-blood ratio. The plasma-to-blood ratios, given in S2B Fig, were included in the kinetic analysis. The plasma-to-blood ratio for 18F-FDG decreased from 1.59 to 1.04 [26]. For 18F-FET, the ratio slightly increased from 1.10 to 1.30. The 18F-FCho ratio was stable over time (0.97 to 1.03).

Metabolites of 18F-FCho. The fraction of lipophilic compounds in plasma was lower than 15% for all time points, see S1 Table. More than 85% of the radioactivity was found in the hydrophilic fraction, which was injected onto the HPLC. One hydrophilic metabolite, assumed to be 18F-FBet, appeared in plasma and amounts to 29.3% 15 min p.i. The fraction of plasma radioactivity contributing to 18F-FCho decreased to 17.2%. Phospho-18F-FCho was detected at low plasma concentrations (2.8% 15 min p.i.). Metabolite correction was applied including a parent fraction curve in the analysis described by a 3-exponential function. The 18F-FBet fraction curve was included applying 3C2i.

Kinetic modeling. Complete results are shown in S4 Fig and Table 1. For a graphical illustration of the results, see S4 Fig.

**Table 1:** Fitting parameters of 18F-FDG, 18F-FET and 18F-FCho in GB and RN.

	AICmean*			SCmean^†^			MSCmean^‡^			K1 (ml/ccm/min)	k2 (min^-1^)	k3 (min^-1^)	Vd^§^ (ml/ccm)	Vt ^ǁ^ (ml/ccm)	Ki^£^ (ml/ccm/min)	Ki ^¶^ (ml/ccm/min)	Kp^#^	SE** K1 (%)	SE** k2 (%)	SE** k3 (%)
18F-FDG	^§§^1C1i	2C1i	3C2i	1C1i	2C1i	3C2i	1C1i	2C1i	3C2i	2C1i	2C1i	2C1i	1C1i	Logan	2C1i	Patlak		2C1i	2C1i	2C1i
GB^††^		28.28			33.34			3.10		0.16	0.19	0.07			0.04	0.04	0.26	4.87	12.50	10.00
RN^‡‡^		78.88			83.94			1.09		0.17	0.28	0.04			0.02	0.02	0.12	7.10	13.72	13.88
18F-FET	^§§^1C1i	2C1i	3C2i	1C1i	2C1i	3C2i	1C1i	2C1i	3C2i	1C1i	1C1i		1C1i	Logan	2C1i	Patlak		1C1i	1C1i	
GB^††^	30.77	72.01		34.10	104.07		2.64	1.59		1.09	0.05		1.09	1.09	0.01	0.02		3.09	5.32	
RN^‡‡^	104.07	103.70		75.88	108.65		3.02	1.05		0.53	0.05		0.53	0.52	0.00	0.01		8.39	15.8	
18F-FCho	^§§^1C1i	2C1i	3C2i	1C1i	2C1i	3C2i	1C1i	2C1i	3C2i	1C1i	1C1i		1C1i	Logan	2C1i	Patlak		1C1i	1C1i	
GB^††^	93.89	92.09	145.37	96.39	94.78	119.59	0.20	0.09	-1.90	0.11	0.05		2.33	1.02	0.02	0.05		10.77	145.16	
RN^‡‡^	91.45	88.56	149.01	93.94	91.31	122.50	0.34	0.49	-1.76	0.10	0.07		1.43	0.96	0.05	0.05		15.61	57.94	

^††^ Glioblastoma (GB),

^‡‡^ Radiation necrosis (RN),

* Akaike information criterion (AIC),

^†^ Schwartz criterion (SC),

^‡^ Model selection criterion (MSC),

^§^ Distribution volume (Vd),

^ǁ^ Total distribution volume (Vt),

^£^ Influx rate (Ki),

^¶^ Slope of the Patlak curve-metabolic flux (Ki),

^#^ Phosphorylation rate (Kp),

** Standard error (SE),

^§§^ 1 compartmental model 1 input function (1C1i)—2 compartmental model 1 input function (2C1i)—3 compartmental model 2 input functions (3C2i).

18F-FDG. S4A Fig. Plasma glucose level was 3.44 mmol/l. Mean k_2_ was significantly higher in RN (0.28±0.03 min^-1^) than in GB (0.19±0.04 min^-1^) while mean k_3_ was significantly higher in GB (0.07±0.03 min^-1^) than in RN (0.04±0.01 min^-1^) (p = 0.009 and p = 0.017, respectively). K_1_ was not significantly different between GB and RN (p = 0.662) and k_4_ = 0 min^-1^ gave optimal SE values. Both K_p_ and K_i_ were significantly higher in GB compared to RN (p = 0.004 and p = 0.030, respectively).

18F-FET. S4B Fig. AIC and SC values were significantly different applying 1C1i compared to 2C1i in GB (both p = 0.05), while no significant difference was found in RN (p = 0.917 and p = 0.754, respectively). Equally, the MSC was only significantly different between 1C1i and 2C1i in GB (p = 0.05). In the RN group 1 MSC outlier value was detected. SEs were as low as possible applying 1C1i, while high values of k_3_ SE (%) were found applying 2C1i (not shown). Based on these observations, 1C1i was selected (Table 1). Data from 2 GB rats was excluded because of unreliable data; in one animal due to a blood clot in the venous catheter influencing the AIF and in another lesion due to a complete mismatch of the fitted TAC and the data points in Pmod. K_1_ was significantly higher in GB (0.06±0.01 ml/ccm/min) compared to RN (0.02±0.01 ml/ccm/min) (p = 0.036). k_2_ was not significantly different in GB (0.05±0.01 min^-1^) compared to RN (0.05±0.01 min^-1^) (p = 0.250). Mean V_d_ was significantly higher in GB (1.09±0.18 ml/ccm) than in RN (0.53±0.10 ml/ccm) (p = 0.036).

18F-FCho. S4C Fig Visual analysis of the fitted curves revealed comparable fits applying 1C1i and 2C1i while bad fits were obtained applying 3C2i (not shown). No significant different AIC, SC and MSC values were found between 1C1i and 2C1i in GB and RN. Applying 3C2i, higher AIC and lower MSC were obtained compared to 1C1i/2C1i (Table 1). SEs were as low as possible applying 1C1i, while high outlier values were found for k_3_ and k_4_ applying 2C1i and 3C2i, both in GB and RN. As such, 1C1i was selected. K_1_ (0.11±0.03 ml/ccm/min in GB and 0.10±0.02 ml/ccm/min in RN) and K_2_ (0,05±0,02 min^-1^ in GB and 0,07±0,01 min^-1^ in RN) were not significantly different between GB and RN (p = 0.530 and 0,251, respectively). Consequently, V_d_ was not significantly different between GB (2.33±1.07 ml/ccm) and RN (1.43±0.37 ml/ccm) (p = 0.117).

Graphical analysis. Complete results are shown in S4 Fig and Table 1. For a graphical illustration of the results, see S4 Fig.

18F-FDG. S4A Fig. Visually, a good fit was obtained by Patlak GA. K_i_ was significantly higher in GB (0.04±0.01 ml/ccm/min) compared to RN (0.02±0.01 ml/ccm/min) (p = 0.017), visible on the parametric maps (row 1, Fig 3). K_i_ (2C1i) was strongly correlated with the Patlak slope with a Spearman correlation coefficient of 0.98 (p = 0.01).

18F-FET. S4B Fig. Assuming that 18F-FET is a reversible bound tracer, Logan GA was selected. An acceptable visual fit and SEs were obtained. V_t_ was significantly higher in GB (1.09±0.20 ml/ccm) than in RN (0.52±0.06 ml/ccm) (p = 0.036). Parametric images showed a heterogeneous increased V_t_ in GB. In RN, V_t_ was slightly increased but showed a more homogeneous distribution (row 2, Fig 3). V_d_ and V_t_ were strongly correlated, with comparable values (Spearman’s rho = 0.881).

18F-FCho. S4C Fig. Both Patlak and Logan GA were performed. Visually, a better fit was obtained using Logan GA. However, the SEs of K_i_ were lower than the SEs of V_t_. Both K_i_ (0.05±0.02 ml/ccm/min in GB and 0.05±0.01 ml/ccm/min in RN) and V_t_ (1.02±0.26 ml/ccm in GB and 0.96±0.20 ml/ccm in RN) were not significantly different between both groups (p = 0.465 and 0.602, respectively) (Table 1). Parametric images of V_t_ showed increased values within GB and RN compared to normal brain tissue (row 3, Fig 3). K_i_ (2C1i) and V_d_ (1C1i) were not correlated with K_i_ and V_t_, with a Spearman’s rho of 0.042 and 0.006, respectively.

#### Correlation between semi-quantitative and quantitative analysis

Spearman correlations (R_s_) between the kinetic/graphical measures and SUV variables were calculated for each tracer. R_s_ and p values can be found in Table 2.

**Table 2:** Spearman correlation coefficients (Rs) between kinetic/graphical measures and SUV.

		SUV_max_		SUV_mean_	
		R_s_*	p	R_s_*	p
18F-FDG_early_	K_1_	0.509	0.110	0.818	0.002
	k_2_	-0.555	0.011	-0.282	0.401
	k_3_	0.765	0.006	0.565	0.070
	K_p_^#^	0.791	0.004	0.564	0.071
	K_i_^£^	0.873	<0.001	0.791	0.004
	K_i Patlak_^¶^	0.900	<0.001	0.755	0.007
18F-FDG_late_	K_1_	0.406	0.244	0.503	0.138
	k_2_	-0.661	0.038	-0.685	0.029
	k_3_	0.663	0.037	0.523	0.121
	K_p_^#^	0.806	0.005	0.697	0.025
	K_i_^£^	0.673	0.033	0.782	0.008
	K_i Patlak_^¶^	0.733	0.016	0.782	0.008
18F-FET	K_1_	0.838	0.009	0.838	0.009
	k_2_	0.855	0.007	0.855	0.007
	V_d_^§^	0.024	0.955	0.024	0.955
	V_t Logan_^ǁ^	-0.024	0.955	-0.024	0.955
18F-FCho	K_1_	0.207	0.567	-0.085	0.815
	k_2_	0.261	0.467	0.067	0.854
	V_d_^§^	0.006	0.987	-0.085	0.815
	V_t Logan_^ǁ^	0.273	0.446	0.006	0.987

* Spearman correlation coefficients (Rs),

^#^ Phosphorylation rate (Kp),

^£^ Influx rate (Ki),

^¶^ Slope of the Patlak curve-metabolic flux (Ki),

^§^ Distribution volume (Vd),

^ǁ^ Total distribution volume (Vt).

**Fig 4:**
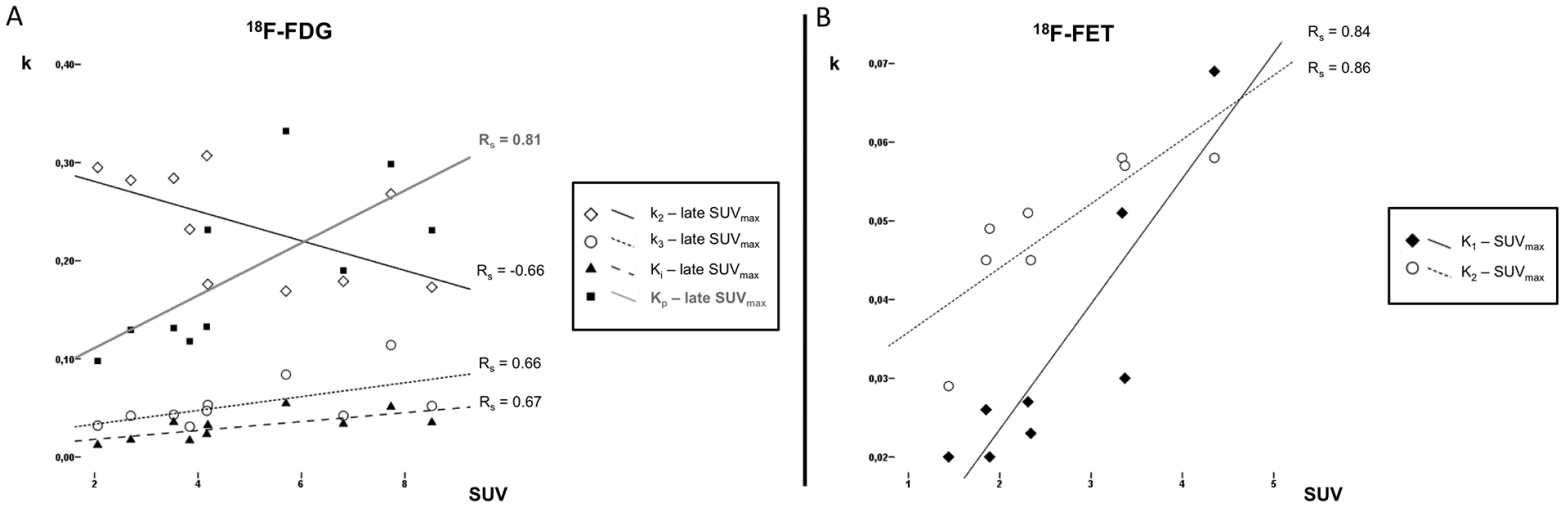
Spearman correlation (Rs) scatterplots between kinetic parameters and SUV_max_ for late 18F-FDG and 18F-FET PET. Late 18F-FDG SUV_max_ is negatively correlated with k_2_ and positively correlated with k_3_, K_i_ and K_p_ (A). 18F-FET SUV_max_ is positively correlated with K_1_ and k_2_ (B).

18F-FDG. Both on early and late 18F-FDG PET k_2_ was significantly negatively correlated with SUV_max_, LNR_mean_, and LNR_max_ (p = 0.038/0.002/0.016 and p = 0.011/0.010/0.015). K_3_ was significantly positively correlated with SUV_max_, both on the early (p = 0.037) and late (p = 0.006) scan. SUV_max_ was also correlated with K_i_ and K_p_ on the early (p = <0.001/0.004) and late (p = 0.033/0.005) 18F-FDG PET. Scatterplots of late SUV_max_ correlations can be found in Fig 4A.

18F-FET. SUV_max_, SUV_mean_, LNR_mean_ and LNR_max_ were significantly positively correlated with K_1_ (p = 0.009/0.009/0.031/0.002) and k_2_ (p = 0.007/0.007/0.003/0.022). Scatterplots of SUV_max_ correlations can be found in Fig 4B.

18F-FCho. No significant correlations were found between 18F-FCho SUV variables and kinetic/graphical parameters.

## Discussion

The potential of 18F-FDG and 18F-FET SUV analysis for discriminating GB and RN was confirmed in our previous publication. SUV values of 18F-FCho PET were not able to differentiate GB and RN [9]. To clarify those results, in this study, we explored the uptake mechanisms of the three tracers using KM and GA. KM is the most accurate method to analyze PET but required ABS and AIF corrections [14]. No metabolite correction was applied for 18F-FDG and 18F-FET because both are metabolically relatively stable *in vivo*. The percentage of intact FET of total plasma radioactivity is 95% 5 min and 87% 120 min p.i., indicating that the fraction of metabolites is low [8]. For 18F-FCho, metabolite correction was necessary due to the known oxidation of choline in hepatocytes and nephrocytes to betaine, which is an important donor of methyl groups for synthesis of methionine and serves as an osmolyte (S3 Fig) [27,32,33]. This side reaction complicates data interpretation of 18F-FCho PET.

A major factor influencing uptake in RN and GB is passive leakage through a damaged BBB, present in GB due to its aggressive nature and in RN due to irradiation endothelial cell damage [34]. This will influence K1, which accounts for both the transport of the tracer from the blood to the interstitial space and the uptake from the interstitial space into the cell by certain transporters. However, K1 is not able to differentiate between passive leakage and uptake by a transport mechanism [35]. When a 2C1i was applied to model uptake of 18F-FDG in GB and RN, k_4_ was set to zero [15]. K_1_ was not significantly different between GB and RN. Therefore, we cannot conclude a higher amount of GLUT in GB compared to RN. A major consideration is that the contribution of passive diffusion of 18F-FDG due to BBB breakdown is unknown. However we could hypothesize that the K1 in GB influenced by the cerebral blood flow, cerebral blood volume, GLUT transport and disrupted BBB is somehow comparable to the K1 in RN predominantly influenced by severe BBB disruption. Phosphorylation of 18F-FDG by hexokinase (k_3_/K_p_) was significantly higher in GB compared to RN while outward transportation of non-phosphorylated tracer (k_2_) was higher in RN than in GB. This implies a higher influx rate in GB. Using Patlak analysis, K_i_ was also higher in GB. SUV_max_, was positively correlated with k_3_, K_p_, K_i_ and the Patlak slope, both using the dynamic or the delayed 18F-FDG imaging protocol (Table 2). A negative correlation was found between SUV_max_ and k_2_, fitting the assumed 2C1i model. This could mean that the differences in SUV_max_ could be explained by differences in both k_2_ and k_3_. A higher k_2_ in RN compared to GB could be explained by the severe vascular damage leading to a faster wash-out. Based on these results more trapping of 18F-FDG occurs in GB and quantification is possible using KM or GA, however, GA is not able to discriminate K_1_ from k_3_ [14].

Based on the SEs, 1C1i was selected quantifying 18F-FET uptake in GB and RN. This was assumed due to the Na^+-^independent route of FET transport via LAT and the Na^+-^ dependent activity via system B^0,+^ and B^0^. Since large neutral amino acids enter normal brain tissue, disruption of BBB is not a prerequisite for intratumoral 18F-FET accumulation [8, 36]. K_1_, V_d_ and V_t_ were significantly higher in GB compared to RN, probably by the presence of more L, B^0,+^ and B^0^ transporters in GB. Clinical results in different tumor types indicate that transport mechanisms of FET may be more complex and one may speculate that FET is selectively transported by LAT2 [10]. A different K_1_ between GB and RN might also relate to differences in cerebral blood flow and cerebral blood volume, which is known to be higher in GB due to neoangiogenesis. An equal amount of 18F-FET efflux (k_2_) between GB, mediated by the LAT antiporter and partially leakage, and RN, mediated by mainly leakage, was shown. Both K_1_ and k_2_ were positively correlated with SUV_max_, which could be explained by the antiporter function of the amino-acid transporter. The significant correlations found between SUV and quantitative measures could indicate that SUV captures the differences in K_1_ and k_2_ (Table 2). Though KM allowed the absolute quantification and better characterization of 18F-FDG and 18F-FET uptake in GB and RN, looking at the TACs in S2 Fig and based on our previous results [9], it appears that a static image 40–60 min p.i. was able to differentiate GB and RN as well as the parametric images derived from the kinetic analysis.

2C1i was expected for quantifying 18F-FCho uptake due to the presumed phosphorylation of 18F-FCho with subsequent incorporation in cellular membranes [21,22]. The percentage 18F-FCho in plasma decreased rapidly concomitant with the appearance of a hydrophilic metabolite, most likely 18F-FBet (S3 Fig). Phosphatidylcholine is the most important metabolite of choline. In the mitochondria of liver and kidney choline is oxidized to betaine [27]. Choline is also a precursor of the neurotransmitter acetylcholine. Acetylcholine could also be synthesized and released from nonneuronal cells, however, its role outside of neurons is not clearly defined [33]. Uptake of choline radiotracers by muscle is low, however, an increase has been shown by Bansal et al. over the first 20 min post-injection, most likely reflecting uptake of betaine metabolites [27]. However, the percentage of total plasma radioactivity attributed to 18F-FBet was lower compared to the literature [22,27,32]. Verwer et al. suggested that this fast metabolization could play a role in the inability of KM to accurately distinguish between the first compartment (K_1_ and k_2_) and signal originating from blood volume in the VOI and between K_1_ and k_3_ [12]. It was also suggested that K_1_, partly determined by angiogenesis and microvessel density, and not the phosphorylation (k_3_) was the key factor for choline uptake [21]. This is in line with the assumption that a static uptake value 1 h p.i. could be reliable for choline kinetics due to the involvement of choline transport in the first minutes, whereas CK gets involved mostly in the later time period [21]. These assumptions are in favor of a 1C1i model. Recently, 1C1i with metabolite-corrected AIF was proposed for quantifying 18F-FCho uptake in prostate cancer [12]. In this study, quantification of 18F-FCho uptake reached optimal fitting using a reversible model. However, K_1_ and V_t_ were not significantly different between GB and RN. As such, we cannot conclude an increased choline transporter-like proteins mediated transport or a higher expression of CK in GB compared to RN. Unfortunately, due to the inability of differentiating K_1_ and k_3_, the amount of 18F-FCho uptake mediated by leakage through the damaged BBB or active trapping was not possible. Only uptake by passive diffusion was negligible due to its polar characteristics [21]. Furthermore, the immediate metabolization raises the question if leakage of 18F-FBet through the damaged BBB played a role. Probably a more metabolically stable 18F-labeled choline tracer would overcome this fast oxidation problem [37]. As such, although 18F-FCho seemed promising for differentiation of tumor recurrence and RN in the clinic [38,39], this was not confirmed in our pre-clinical study. However, we should keep in mind that the metabolism of choline tracers in humans is slower than in rodents and that the uptake was only investigated in one glioma model [12,26,32,37].

## Conclusion

Using a 2C1i model more trapping of 18F-FDG (k3) was found in GB compared to RN. Secondly, the influx of 18F-FET was higher in GB compared to RN using a 1C1i model. Important correlations were found between SUV and kinetic or graphical measures for 18F-FDG and 18F-FET. Based on these results we assume that SUV is able to capture kinetic differences. For 18F-FCho, a 1 compartmental model is suggested for absolute quantification, but does not allow clarifying the uptake mechanism in GB and RN.

## Supporting Information

S1 Fig Contrast-enhanced MRI and semi-quantitative PET analyses of glioblastoma (GB) and radiation necrosis (RN). For clarity, the brain is contoured in white. 18F-FDG PET 40–60 min postinjection (A-B-C) and 240 min postinjection (D-E-F). 18F-FET PET 35–55 min postinjection (G-H-I) and 18F-FCho PET 10–20 min postinjection (J-K-L).
(PDF)

S2 Fig Blood curves and time activity curves. Whole blood and dispersion corrected blood curve (A), plasma-blood ratios (B) and time activity curves (TACs) (C) of 18F-FDG, 18F-FET and 18F-FCho in GB and RN.
(PDF)

S3 Fig Metabolization pattern of 18F-FCho. 18F-FCho enters the cell by CTL, is mainly phosphorylated by CK forming PC which, in turn, will be converted to PPC by Pcyt and CPT. PPC is a major constituent of the cellular membrane. In mitochondria of the liver and kidneys, choline is oxidized to betaine by CD and BAD, which enters the one-carbon cycle and serves as a methyl donor in the remethylation of homocysteine to methionine.
(PDF)

S4 Fig Kinetic modeling and graphical analysis of 18F-FDG (A), 18F-FET (B) and 18F-FCho (C) in GB and RN. Schematic diagram of the selected compartmental model (left) and fitting parameters, kinetic constants and Patlag/Logan plot (right).
(PDF)

S1 Table Percentage of 18F-FCho metabolites present in arterial plasma. (PDF)
