# Correction: Kinetic Modeling and Graphical Analysis of 18F-Fluoromethylcholine (FCho), 18F-Fluoroethyltyrosine (FET) and 18F-Fluorodeoxyglucose (FDG) PET for the Fiscrimination between High-Grade Glioma and Radiation Necrosis in Rats

**DOI:** 10.1371/journal.pone.0164208

**Published:** 2016-10-03

**Authors:** Julie Bolcaen, Kelly Lybaert, Lieselotte Moerman, Benedicte Descamps, Karel Deblaere, Tom Boterberg, Jean-Pierre Kalala, Caroline Van den Broecke, Filip De Vos, Christian Vanhove, Ingeborg Goethals

The word “Discrimination” is misspelled in the article title. The correct title is: Kinetic Modeling and Graphical Analysis of 18F-Fluoromethylcholine (FCho), 18F-Fluoroethyltyrosine (FET) and 18F-Fluorodeoxyglucose (FDG) PET for the Discrimination between High-Grade Glioma and Radiation Necrosis in Rats.

## References

[1] Bolcaen J, Lybaert K, Moerman L, Descamps B, Deblaere K, Boterberg T, et al (2016) Kinetic Modeling and Graphical Analysis of 18F-Fluoromethylcholine (FCho), 18F-Fluoroethyltyrosine (FET) and 18F-Fluorodeoxyglucose (FDG) PET for the Fiscrimination between High-Grade Glioma and Radiation Necrosis in Rats. PLoS ONE 11(8): e0161845 doi:10.1371/journal.pone.0161845 27559736 10.1371/journal.pone.0161845 PMC4999092

