# Supplementary material for: Kinetic Modeling and Graphical Analysis of 18F-Fluoromethylcholine (FCho), 18F-Fluoroethyltyrosine (FET) and 18F-Fluorodeoxyglucose (FDG) PET for the Fiscrimination between High-Grade Glioma and Radiation Necrosis in Rats
Source: PLoS One. 2016 Aug 25;11(8):e0161845. doi: 10.1371/journal.pone.0161845 (PMC4999092; doi:10.1371/journal.pone.0161845)
Supplement: S1 Fig — For clarity, the brain is contoured in white. 18F-FDG PET 40–60 min postinjection (A-B-C) and 240 min postinjection (D-E-F). 18F-FET PET 35–55 min postinjection (G-H-I) and 18F-FCho PET 10–20 min postinjection (J-K-L). (PDF) [file pone.0161845.s001.pdf]

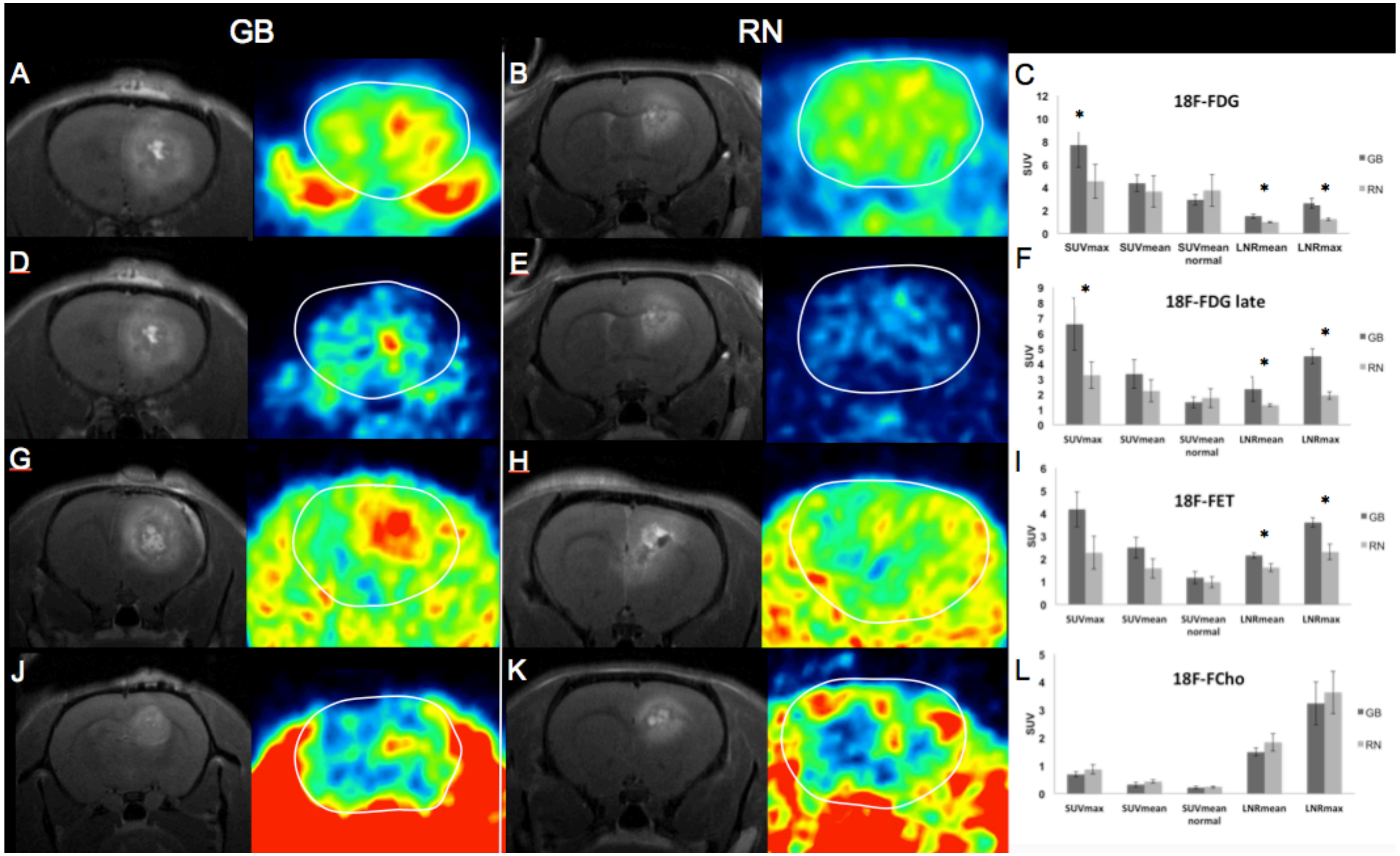

S1 Fig. Contrast-enhanced MRI and semi-quantitative PET analyses of glioblastoma (GB) and radiation necrosis (RN). For clarity, the brain is contoured in white. 18F-FDG PET 40–60 min postinjection (A-B-C) and 240 min postinjection (D-E-F). 18F-FET PET 35-55 min postinjection (G-H-I) and 18F-FCho PET 10-20 min postinjection (J-K-L).
