# Supplementary material for: Kinetic Modeling and Graphical Analysis of 18F-Fluoromethylcholine (FCho), 18F-Fluoroethyltyrosine (FET) and 18F-Fluorodeoxyglucose (FDG) PET for the Fiscrimination between High-Grade Glioma and Radiation Necrosis in Rats
Source: PLoS One. 2016 Aug 25;11(8):e0161845. doi: 10.1371/journal.pone.0161845 (PMC4999092; doi:10.1371/journal.pone.0161845)
Supplement: S2 Fig — Whole blood and dispersion corrected blood curve (A), plasma-blood ratios (B) and time activity curves (TACs) (C) of 18F-FDG, 18F-FET and 18F-FCho in GB and RN. (PDF) [file pone.0161845.s002.pdf]

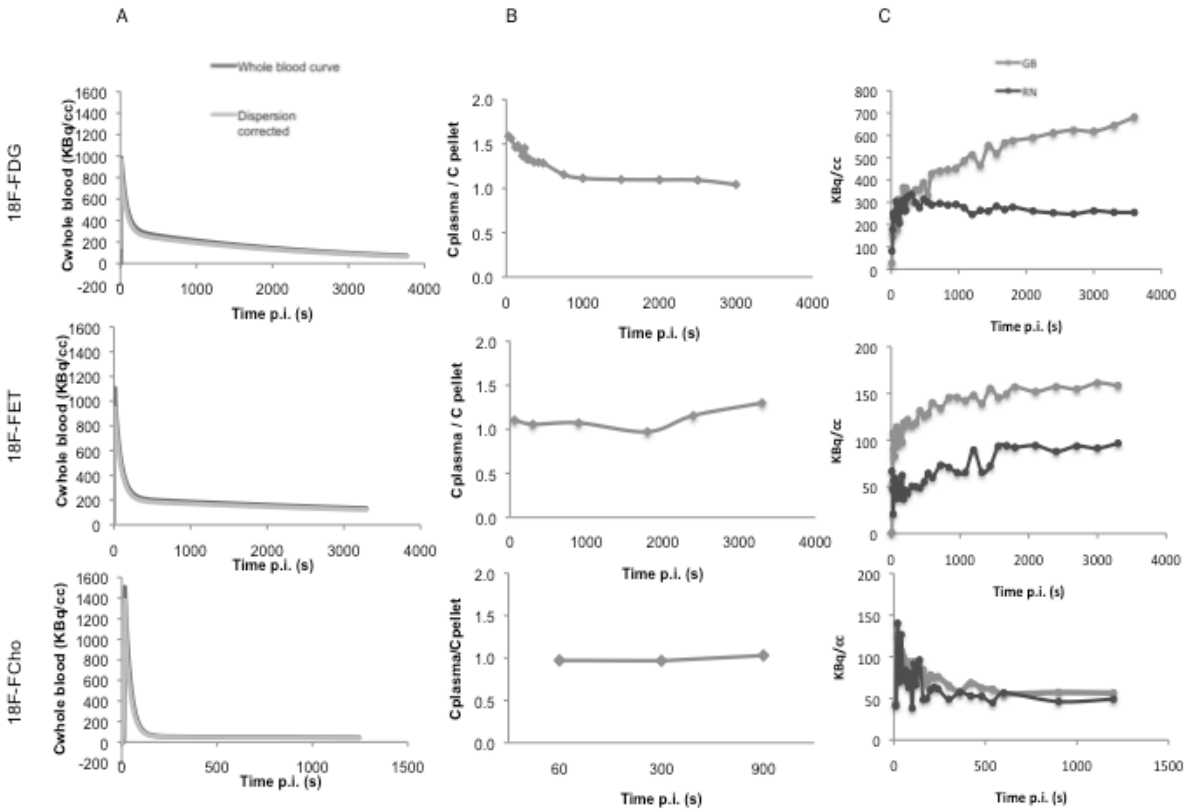

S2 Fig. Blood curves and time activity curves. Whole blood and dispersion corrected blood curve (A), plasma-blood ratios (B) and time activity curves (TACs) (C) of 18F-FDG, 18F-FET and 18F-FCho in GB and RN.
