# Supplementary material for: Kinetic Modeling and Graphical Analysis of 18F-Fluoromethylcholine (FCho), 18F-Fluoroethyltyrosine (FET) and 18F-Fluorodeoxyglucose (FDG) PET for the Fiscrimination between High-Grade Glioma and Radiation Necrosis in Rats
Source: PLoS One. 2016 Aug 25;11(8):e0161845. doi: 10.1371/journal.pone.0161845 (PMC4999092; doi:10.1371/journal.pone.0161845)
Supplement: S3 Fig — 18F-FCho enters the cell by CTL, is mainly phosphorylated by CK forming PC which, in turn, will be converted to PPC by Pcyt and CPT. PPC is a major constituent of the cellular membrane. In mitochondria of the liver and kidneys, choline is oxidized to betaine by CD and BAD, which enters the one-carbon cycle and serves as a methyl donor in the remethylation of homocysteine to methionine. (PDF) [file pone.0161845.s003.pdf]

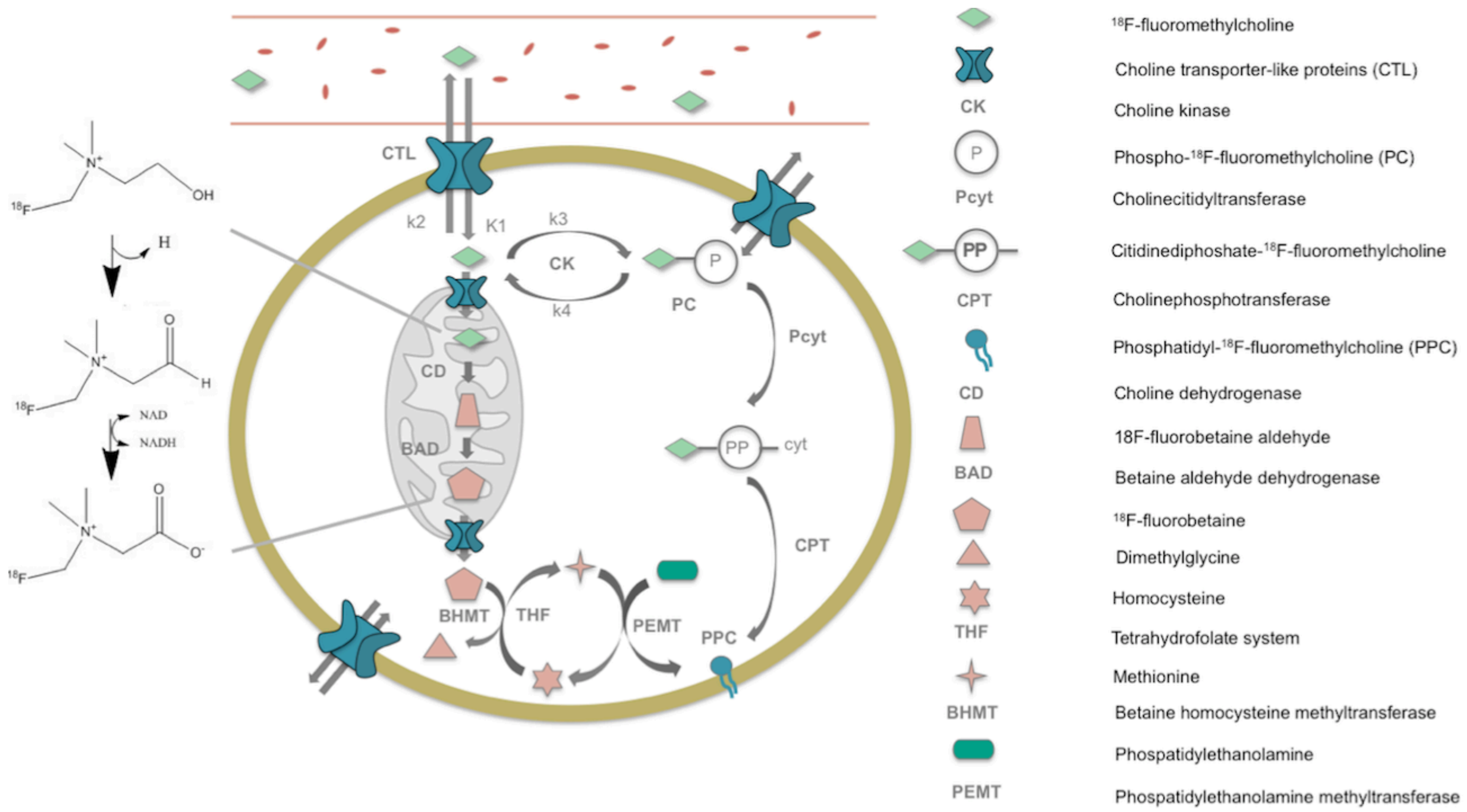

S3 Fig. Metabolization pattern of ^{18}F-FCho. ^{18}F-FCho enters the cell by CTL, is mainly phosphorylated by CK forming PC which, in turn, will be converted to PPC by Pcyt and CPT. PPC is a major constituent of the cellular membrane. In mitochondria of the liver and kidneys, choline is oxidized to betaine by CD and BAD, which enters the one-carbon cycle and serves as a methyl donor in the remethylation of homocysteine to methionine.
