# Supplementary material for: Kinetic Modeling and Graphical Analysis of 18F-Fluoromethylcholine (FCho), 18F-Fluoroethyltyrosine (FET) and 18F-Fluorodeoxyglucose (FDG) PET for the Fiscrimination between High-Grade Glioma and Radiation Necrosis in Rats
Source: PLoS One. 2016 Aug 25;11(8):e0161845. doi: 10.1371/journal.pone.0161845 (PMC4999092; doi:10.1371/journal.pone.0161845)
Supplement: S4 Fig — Schematic diagram of the selected compartmental model (left) and fitting parameters, kinetic constants and Patlag/Logan plot (right). (PDF) [file pone.0161845.s004.pdf]

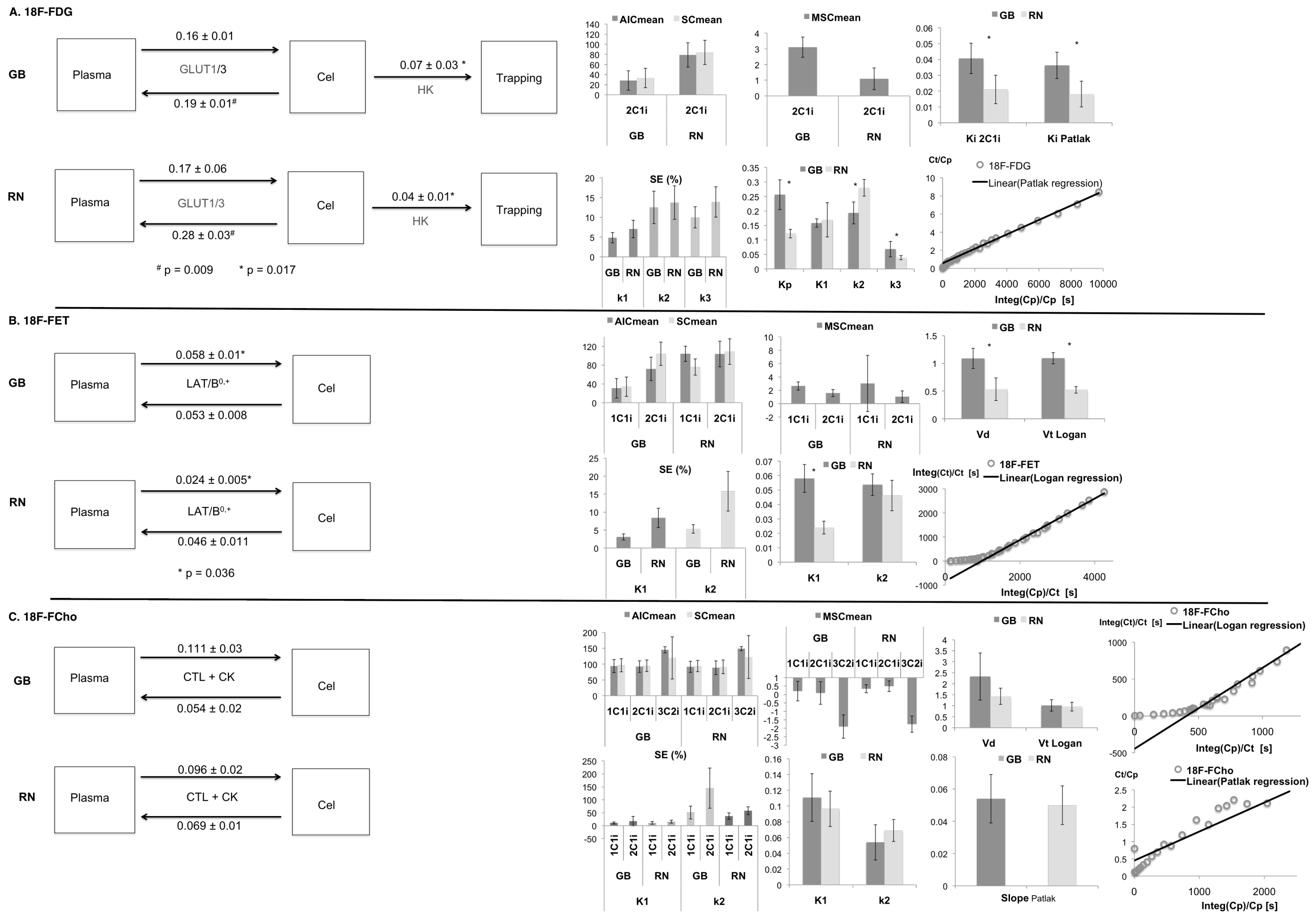

S4 Fig. Kinetic modeling and graphical analysis of ^{18}F-FDG (A), ^{18}F-FET (B) and ^{18}F-FCho (C) in GB and RN. Schematic diagram of the selected compartmental model (left) and fitting parameters, kinetic constants and Patlag/Logan plot (right).
