# Supplementary material for: Kinetic Modeling and Graphical Analysis of 18F-Fluoromethylcholine (FCho), 18F-Fluoroethyltyrosine (FET) and 18F-Fluorodeoxyglucose (FDG) PET for the Fiscrimination between High-Grade Glioma and Radiation Necrosis in Rats
Source: PLoS One. 2016 Aug 25;11(8):e0161845. doi: 10.1371/journal.pone.0161845 (PMC4999092; doi:10.1371/journal.pone.0161845)
Supplement: S1 Table — (PDF) [file pone.0161845.s005.pdf]

Time p.i. (min)	Hydrophilic metabolites (%)	Lipophilic metabolites (%)	18F-FCho (%)	18F-FBet (%)	Phospho-^{18}F-FCho (%)	Other metabolites (%)
0	0	0	100	0	0	0
1	86.7 ± 0.5	13.3 ± 0.5	81.1	3.4	0.4	15.2
5	93.4 ± 1.9	6.6 ± 1.9	62.4	2.7	2.6	32.3
15	93.4 ± 0.5	6.7 ± 0.5	17.2	29.3	2.8	21.4

S4 Table. Percentage of 18F-FCho metabolites present in arterial plasma.
